# Supplementary material for: A Likelihood Approach for Real-Time Calibration of Stochastic Compartmental Epidemic Models
Source: PLoS Comput Biol. 2017 Jan 17;13(1):e1005257. doi: 10.1371/journal.pcbi.1005257 (PMC5240920; doi:10.1371/journal.pcbi.1005257)
Supplement: S1 File — (TAR.GZ) [file pcbi.1005257.s014.tar.gz › HSPH_Online-SI-Revision/PF08/n1-extreme/n1-extreme_PF_table-8.pdf]

1	R0	{2.95019, 14.2854}	{1.94918, 11.5058}	{{1.27357, 5.3704}, {2.63959, 17.3398}}	{33.9305, 29.8094}	inf	3.	38.5574	{2.59214, 90.7609}	1197.24	0.946045	newly	0.1	44.86	{2., 102.}	44770.	2.22719	Reff	2.93839	1.78035	{1.18566, 2.48668}	39.4106	ar	924.	660.1	{233., 902.}	28.6991
2	R0	{2.67565, 16.946}	{1.78944, 12.9643}	{{1.07874, 6.31117}, {2.73737, 18.2815}}	{34.5306, 27.4813}	inf	30.	75.5003	{6.98202, 164.688}	187.011	0.469283	newly	15.	44.01	{5., 90.}	220.6	0.485132	Reff	2.55525	1.34496	{0.872175, 2.0885}	47.3647	ar	926.	615.42	{197., 903.}	33.7235
3	R0	{2.40066, 16.6543}	{1.9221, 13.4072}	{{1.12882, 6.55128}, {2.79269, 19.0443}}	{26.7693, 27.0272}	inf	14.	56.9511	{4.78513, 139.11}	337.819	0.585315	newly	8.	36.28	{3., 89.}	380.	0.60466	Reff	2.34544	1.62524	{1.04836, 2.23343}	32.6008	ar	916.	653.13	{137., 917.}	28.9618
4	R0	{1.9294, 15.4361}	{1.85189, 13.2782}	{{1.10693, 6.62607}, {2.73737, 18.8445}}	{25.2646, 23.3404}	inf	10.	61.1711	{3.8551, 173.588}	530.42	0.688134	newly	5.	35.34	{3., 82.}	622.8	0.773984	Reff	1.89467	1.525	{0.961356, 2.17624}	26.0768	ar	796.	648.69	{236., 913.}	26.7349
5	R0	{2.92859, 19.5393}	{1.71004, 14.1592}	{{1.22664, 7.17948}, {2.41054, 20.}}	{41.7799, 28.5203}	inf	28.	86.9102	{20.9301, 199.477}	222.195	0.427109	newly	16.	41.03	{13., 83.}	163.563	0.358099	Reff	2.7968	1.099	{0.743688, 1.60181}	60.705	ar	927.	745.37	{491., 1009.}	23.3732
6	R0	{1.79622, 12.3148}	{1.83091, 13.2997}	{{1.05743, 6.88118}, {2.73517, 17.5057}}	{27.9422, 28.0586}	inf	35.	91.0996	{7.16405, 212.929}	197.584	0.514908	newly	31.	47.73	{6., 97.}	100.871	0.435418	Reff	1.6651	1.23133	{0.807409, 1.70495}	31.8937	ar	734.	637.3	{214., 907.}	28.7275
7	R0	{2.45855, 17.1776}	{1.99968, 12.3906}	{{1.20105, 6.01426}, {2.80569, 18.9248}}	{24.7011, 31.5064}	inf	24.	50.5127	{2.57459, 120.356}	158.227	0.529472	newly	13.	43.06	{1., 108.}	273.846	0.636774	Reff	2.38971	1.75007	{1.12756, 2.46614}	29.0756	ar	871.	664.97	{69., 928.}	26.3502
8	R0	{2.20009, 15.519}	{1.67046, 13.3465}	{{1.11228, 5.32461}, {2.24606, 19.0269}}	{28.8429, 27.6262}	inf	26.	71.5845	{2.14702, 167.7}	217.95	0.598465	newly	18.	36.74	{1., 77.}	144.444	0.515565	Reff	2.00208	1.14718	{0.783675, 1.56433}	44.0022	ar	839.	598.97	{256., 862.}	30.5113
9	R0	{2.06493, 9.6414}	{1.95969, 11.5128}	{{1.27357, 5.3704}, {2.63959, 17.3398}}	{22.2057, 40.9513}	inf	4.	38.4091	{2.90181, 90.7609}	873.104	0.841649	newly	3.	42.72	{2., 101.}	1338.67	0.92324	Reff	2.04222	1.79145	{1.18566, 2.48668}	22.5744	ar	777.	672.37	{263., 903.}	23.9704
10	R0	{2.60775, 19.6936}	{1.62901, 12.9082}	{{1.11365, 5.98216}, {2.21411, 18.9813}}	{38.0524, 34.5298}	inf	29.	61.9917	{2.57939, 140.409}	161.703	0.519172	newly	16.	33.61	{2., 69.}	151.063	0.496682	Reff	2.47997	1.11601	{0.758662, 1.56866}	55.3014	ar	889.	583.92	{242., 831.}	34.5962
11	R0	{1.99168, 8.13555}	{1.99968, 12.3906}	{{1.20105, 6.01426}, {2.80569, 18.9248}}	{24.5245, 63.3446}	inf	4.	50.5127	{2.57459, 120.356}	1177.98	0.947098	newly	3.	43.06	{1., 108.}	1353.33	0.986634	Reff	1.97375	1.75007	{1.12756, 2.46614}	24.3419	ar	772.	664.97	{69., 928.}	27.6516
12	R0	{1.79304, 9.18941}	{1.94918, 11.5058}	{{1.27357, 5.3704}, {2.63959, 17.3398}}	{24.761, 45.6723}	inf	3.	38.5574	{2.59214, 90.7609}	1197.24	0.946045	newly	4.	44.86	{2., 102.}	1040.5	0.848042	Reff	1.78587	1.78035	{1.18566, 2.48668}	21.6138	ar	725.	660.1	{233., 902.}	26.6869
13	R0	{1.84016, 18.3431}	{1.62464, 13.3295}	{{1.11365, 6.0993}, {2.22212, 18.9813}}	{22.1334, 28.9669}	inf	36.	62.3116	{2.14702, 142.6}	127.446	0.51224	newly	12.	33.37	{1., 67.}	213.083	0.564136	Reff	1.71319	1.14068	{0.77592, 1.59714}	35.2895	ar	762.	576.99	{245., 850.}	28.9436
14	R0	{1.94221, 9.85971}	{1.8285, 13.3301}	{{1.07874, 6.8071}, {2.64373, 19.1994}}	{24.5106, 46.1138}	inf	16.	65.414	{3.69964, 176.077}	337.993	0.600848	newly	13.	38.18	{2., 82.}	226.615	0.551419	Reff	1.86646	1.47882	{0.966073, 2.05037}	27.1937	ar	805.	640.68	{171., 898.}	26.8199
15	R0	{1.78578, 12.0541}	{1.73976, 14.8396}	{{1.21838, 9.30376}, {2.3976, 20.}}	{22.0988, 35.1816}	inf	16.	91.6414	{28.1181, 187.534}	473.465	0.652662	newly	14.	45.2	{15., 92.}	225.429	0.430782	Reff	1.68578	1.21458	{0.835768, 1.6786}	30.0696	ar	732.	772.27	{531., 1013.}	22.2964
16	R0	{1.94463, 15.6503}	{1.97502, 12.4744}	{{1.21457, 6.01426}, {2.78218, 18.9248}}	{24.1021, 28.6381}	inf	8.	50.1214	{2.57459, 120.356}	550.608	0.743658	newly	4.	42.47	{1., 108.}	982.75	0.894384	Reff	1.92518	1.72756	{1.12756, 2.43684}	23.8476	ar	757.	662.82	{69., 919.}	27.6301
17	R0	{2.08901, 16.4314}	{1.79354, 13.246}	{{1.11228, 6.19362}, {2.73737, 18.4249}}	{27.6406, 25.2414}	inf	17.	75.023	{6.98202, 159.843}	360.361	0.575238	newly	8.	43.46	{5., 94.}	459.75	0.655978	Reff	2.03887	1.34605	{0.862846, 1.99815}	36.1893	ar	826.	617.44	{197., 903.}	30.0799
18	R0	{2.76419, 18.56}	{1.97229, 12.2726}	{{1.18939, 5.62019}, {2.80569, 18.9406}}	{29.8386, 35.2142}	inf	14.	46.9126	{3.78379, 105.973}	265.528	0.5713	newly	7.	41.41	{3., 101.}	518.143	0.721097	Reff	2.70338	1.73387	{1.09441, 2.4542}	35.9675	ar	920.	653.25	{69., 928.}	29.4054
19	R0	{2.3821, 17.1399}	{1.94297, 11.4712}	{{1.27357, 5.3704}, {2.63959, 17.3398}}	{24.0552, 34.9757}	inf	12.	38.4204	{2.59214, 90.7609}	253.179	0.552934	newly	10.	44.79	{2., 102.}	383.1	0.620353	Reff	2.34637	1.77518	{1.18566, 2.48668}	27.3516	ar	883.	658.55	{233., 902.}	26.6082
20	R0	{2.78314, 19.6127}	{1.67262, 13.9652}	{{1.11228, 6.0993}, {2.24606, 19.0443}}	{40.2942, 28.9733}	inf	70.	69.3229	{2.48733, 160.24}	72.8179	0.520305	newly	32.	35.24	{1., 75.}	73.25	0.471782	Reff	2.44916	1.15927	{0.783675, 1.60898}	53.0004	ar	943.	592.99	{245., 870.}	37.1166
21	R0	{1.85124, 14.6794}	{1.77695, 12.9826}	{{1.07662, 6.18069}, {2.73737, 19.0443}}	{28.9381, 26.2961}	inf	18.	74.2517	{6.295, 168.25}	334.369	0.567036	newly	15.	43.13	{5., 90.}	217.667	0.492038	Reff	1.78274	1.34457	{0.872175, 2.0885}	31.0562	ar	730.	603.31	{175., 905.}	31.9959
22	R0	{2.1028, 14.9852}	{1.79191, 13.3079}	{{1.10693, 6.62607}, {2.55942, 19.0269}}	{25.3673, 23.1787}	inf	26.	60.2174	{3.4726, 155.006}	172.793	0.4955	newly	17.	34.7	{2., 80.}	144.235	0.489956	Reff	2.01659	1.47072	{0.931688, 2.05037}	29.8748	ar	840.	629.05	{207., 889.}	28.3464
23	R0	{1.86601, 10.2157}	{1.99779, 12.4424}	{{1.21457, 6.01426}, {2.79269, 18.9248}}	{25.8901, 40.5199}	inf	8.	50.4513	{2.57459, 120.356}	554.731	0.746898	newly	6.	43.06	{1., 108.}	643.333	0.786674	Reff	1.84548	1.74835	{1.13169, 2.4542}	23.487	ar	747.	669.55	{69., 924.}	27.8648
24	R0	{2.34032, 10.0992}	{1.94918, 11.5058}	{{1.27357, 5.3704}, {2.63959, 17.3398}}	{23.2913, 39.3054}	inf	2.	38.5574	{2.59214, 90.7609}	1836.98	1.09123	newly	1.	44.86	{2., 102.}	4396.	1.32719	Reff	2.3333	1.78035	{1.18566, 2.48668}	26.8911	ar	887.	660.1	{233., 902.}	26.5817
25	R0	{1.91548, 15.3401}	{1.81859, 13.3019}	{{1.10693, 6.62607}, {2.64373, 19.0269}}	{25.1678, 23.0879}	inf	10.	58.714	{3.69964, 150.177}	507.109	0.682571	newly	8.	34.02	{2., 75.}	348.	0.628244	Reff	1.88292	1.50458	{0.961356, 2.17624}	27.012	ar	765.	638.79	{236., 908.}	27.7137
26	R0	{2.75283, 10.6721}	{2.00426, 12.5001}	{{1.21457, 6.01426}, {2.80569, 18.9406}}	{28.5054, 37.7903}	inf	2.	51.0656	{2.57459, 120.356}	2464.47	1.20405	newly	1.	43.29	{1., 108.}	4243.	1.37766	Reff	2.73907	1.75265	{1.13169, 2.46614}	36.0895	ar	926.	670.66	{69., 928.}	27.8272
27	R0	{2.08367, 19.7849}	{1.8046, 13.2914}	{{1.11228, 5.72868}, {2.75598, 19.2972}}	{25.7407, 32.8916}	inf	51.	71.4668	{3.79943, 165.044}	103.055	0.532604	newly	27.	35.89	{3., 76.}	87.4444	0.49384	Reff	1.89822	1.29206	{0.765308, 1.96045}	37.4386	ar	833.	617.97	{219., 913.}	29.569
28	R0	{2.64334, 17.1765}	{1.93545, 13.4971}	{{1.11228, 6.55128}, {2.78218, 19.0269}}	{29.1137, 26.4116}	inf	29.	62.3851	{3.55575, 168.293}	165.406	0.509683	newly	16.	37.88	{3., 89.}	181.25	0.510667	Reff	2.5191	1.62197	{1.03424, 2.23343}	36.3624	ar	934.	657.84	{129., 917.}	29.6167
29	R0	{2.87008, 13.3872}	{1.77766, 13.0972}	{{1.08887, 6.8071}, {2.51743, 18.743}}	{38.227, 27.0275}	inf	6.	73.459	{4.99197, 159.976}	1136.7	0.924865	newly	4.	41.38	{3., 91.}	948.	0.886415	Reff	2.82129	1.3396	{0.904447, 1.91401}	52.548	ar	941.	615.92	{172., 900.}	34.5484
30	R0	{2.06105, 19.6275}	{1.73408, 14.169}	{{1.23694, 7.45108}, {2.54431, 20.}}	{25.773, 28.2764}	inf	103.	87.0966	{25.0107, 179.578}	55.7333	0.321116	newly	49.	41.75	{15., 76.}	44.551	0.247188	Reff	1.55609	1.06328	{0.619528, 1.3752}	34.1734	ar	747.	772.26	{511., 1030.}	22.1205
31	R0	{2.30764, 13.435}	{2.00426, 12.5001}	{{1.21457, 6.01426}, {2.80569, 18.9406}}	{23.2153, 28.332}	inf	14.	51.0656	{2.57459, 120.356}	295.383	0.607876	newly	11.	43.29	{1., 108.}	330.818	0.6629	Reff	2.26841	1.75265	{1.13169, 2.46614}	26.9002	ar	877.	670.66	{69., 928.}	25.8176
32	R0	{2.30565, 11.9237}	{1.97229, 12.2726}	{{1.18939, 5.62019}, {2.80569, 18.9406}}	{24.2132, 32.79}	inf	8.	46.9126	{3.78379, 105.973}	509.04	0.716387	newly	11.	41.41	{3., 101.}	313.545	0.633236	Reff	2.26876	1.73387	{1.09441, 2.4542}	27.4125	ar	853.	653.25	{69., 928.}	27.4361
33	R0	{2.24031, 18.582}	{1.73562, 13.0234}	{{1.08887, 6.77341}, {2.46533, 18.743}}	{27.703, 30.8091}	inf	23.	71.6541	{4.32503, 159.976}	246.599	0.55951	newly	10.	38.79	{3., 85.}	312.7	0.597387	Reff	2.17982	1.30893	{0.889763, 1.87746}	40.9307	ar	864.	599.47	{151., 885.}	32.0359
34	R0	{2.33306, 15.9759}	{1.91337, 13.3377}	{{1.12882, 6.31117}, {2.79269, 19.0443}}	{26.4087, 27.3218}	inf	16.	55.7324	{4.78513, 139.11}	282.179	0.551555	newly	12.	35.72	{3., 88.}	234.667	0.524965	Reff	2.27473	1.62135	{1.04836, 2.23343}	31.277	ar	854.	650.18	{137., 917.}	27.6136
35	R0	{2.48357, 18.3573}	{1.96901, 12.3389}	{{1.20105, 6.01426}, {2.78218, 18.8445}}	{25.3174, 34.1402}	inf	17.	51.1176	{2.57459, 122.239}	236.608	0.567561	newly	9.	42.99	{1., 108.}	412.556	0.703489	Reff	2.43887	1.71812	{1.12479, 2.43684}	30.9692	ar	835.	657.97	{69., 919.}	26.4994
36	R0	{2.59548, 19.9046}	{1.81032, 13.2662}	{{1.07874, 6.62607}, {2.73737, 18.4042}}	{32.4633, 33.3621}	inf	18.	71.1391	{6.72467, 168.25}	317.618	0.542745	newly	13.	41.05	{5., 94.}	243.615	0.505391	Reff	2.50983	1.37779	{0.872175, 2.09254}	45.1115	ar	863.	614.34	{197., 905.}	31.3581
37	R0	{2.29641, 16.9868}	{1.6262, 14.754}	{{1.21861, 7.74337}, {2.16378, 20.}}	{31.3898, 22.1449}	inf	21.	78.1728	{25.036, 163.62}	276.504	0.488347	newly	10.	36.39	{15., 63.}	264.5	0.488915	Reff	2.18159	1.15414	{0.808447, 1.48501}	47.6713	ar	877.	725.84	{470., 957.}	22.0867
38	R0	{2.65743, 15.4569}	{1.75742, 12.9783}	{{1.07662, 6.18069}, {2.73737, 19.0443}}	{35.5532, 26.2479}	inf	29.	71.912	{6.295, 164.688}	185.873	0.478949	newly	16.	41.66	{5., 90.}	193.625	0.473943	Reff	2.52987	1.34067	{0.872175, 2.0885}	47.0063	ar	897.	595.33	{172., 905.}	34.4069
39	R0	{2.67953, 14.5132}	{1.96901, 12.3389}	{{1.20105, 6.01426}, {2.78218, 18.8445}}	{28.3305, 27.6112}	inf	2.	51.1176	{2.57459, 122.239}	2467.07	1.19059	newly	1.	42.99	{1., 108.}	4213.	1.36075	Reff	2.67149	1.71812	{1.12479, 2.43684}	35.901	ar	894.	657.97	{69., 919.}	27.5694
40	R0	{1.94594, 11.928}	{1.79905, 13.4305}	{{1.07874, 6.8071}, {2.52651, 19.1994}}	{24.1552, 30.6278}	inf	31.	61.4808	{3.69964, 160.648}	144.489	0.477914	newly	31.	35.08	{2., 80.}	77.2258	0.467454	Reff	1.84475	1.47539	{0.966073, 2.05037}	26.9982	ar	796.	630.67	{171., 888.}	27.4611
41	R0	{1.66297, 10.0047}	{1.6083, 12.9834}	{{1.07874, 5.32461}, {2.24606, 19.0443}}	{22.5889, 50.404}	inf	25.	60.9562	{2.33643, 132.517}	190.754	0.528927	newly	18.	30.26	{2., 65.}	121.333	0.476105	Reff	1.51995	1.03422	{0.687447, 1.53048}	36.6973	ar	716.	558.59	{184., 831.}	30.4623
42	R0	{2.91568, 16.4453}	{1.94918, 11.5058}	{{1.27357, 5.3704}, {2.63959, 17.3398}}	{33.1498, 33.2934}	inf	9.	38.5574	{2.59214, 90.7609}	355.159	0.617119	newly	8.	44.86	{2., 102.}	490.	0.662906	Reff	2.88652	1.78035	{1.18566, 2.48668}	38.3219	ar	919.	660.1	{233., 902.}	28.346
43	R0	{2.55898, 15.8889}	{1.97502, 12.4744}	{{1.21457, 6.01426}, {2.78218, 18.9248}}	{26.1551, 29.0031}	inf	3.	50.1214	{2.57459, 120.356}	1584.08	1.04614	newly	3.	42.47	{1., 108.}	1333.67	0.981841	Reff	2.54619	1.72756	{1.12756, 2.43684}	32.8704	ar	904.	662.82	{69., 919.}	27.4381
44	R0	{2.28649, 15.9017}	{1.83931, 13.3579}	{{1.10693, 6.74711}, {2.64373, 19.0269}}	{26.5281, 23.4691}	inf	13.	60.1442	{3.69964, 155.006}	386.992	0.617156	newly	10.	35.01	{2., 82.}	276.9	0.571713	Reff	2.24076	1.51632	{0.961356, 2.18291}	33.1887	ar	880.	644.13	{236., 908.}	28.0761
45	R0	{2.65121, 18.6339}	{1.94297, 11.4712}	{{1.27357, 5.3704}, {2.63959, 17.3398}}	{28.2085, 38.8008}	inf	4.	38.4204	{2.59214, 90.7609}	874.589	0.84254	newly	1.	44.79	{2., 102.}	4389.	1.32418	Reff	2.6353	1.77518	{1.18566, 2.48668}	33.2694	ar	928.	658.55	{233., 902.}	29.1476
46	R0	{2.52291, 18.0251}	{1.84646, 13.2556}	{{1.10693, 6.62607}, {2.73737, 18.8445}}	{29.9537, 28.2989}	inf	13.	60.8329	{3.8551, 173.588}	390.859	0.612998	newly	3.	35.01	{3., 82.}	1079.	0.943932	Reff	2.4775	1.51966	{0.961356, 2.17624}	38.8301	ar	894.	645.59	{236., 913.}	28.7864
47	R0	{2.22618, 17.1225}	{1.92206, 13.4182}	{{1.12882, 6.55128}, {2.79269, 19.0443}}	{26.0794, 27.3857}	inf	9.	57.4744	{4.78513, 139.11}	560.506	0.689536	newly	3.	36.57	{3., 89.}	1131.	0.894955	Reff	2.18388	1.62422	{1.04836, 2.23343}	29.2576	ar	850.	652.67	{137., 917.}	27.3047
48	R0	{2.04405, 16.073}	{1.95052, 13.5779}	{{1.12882, 6.55128}, {2.78218, 19.0443}}	{25.2426, 25.2603}	inf	18.	65.932	{3.55575, 174.181}	299.956	0.578419	newly	12.	42.03	{3., 95.}	284.25	0.562209	Reff	1.96229	1.62052	{1.04836, 2.21787}	25.4267	ar	779.	665.41	{129., 914.}	27.2875
49	R0	{2.84396, 18.097}	{1.94297, 11.4712}	{{1.27357, 5.3704}, {2.63959, 17.3398}}	{31.8504, 37.3785}	inf	14.	38.4204	{2.59214, 90.7609}	211.079	0.525046	newly	11.	44.79	{2., 102.}	344.818	0.604624	Reff	2.79846	1.77518	{1.18566, 2.48668}	36.6685	ar	952.	658.55	{233., 902.}	30.8246
50	R0	{1.85341, 18.0975}	{1.95574, 13.0157}	{{1.11164, 6.18069}, {2.78218, 18.9406}}	{28.1272, 30.2188}	inf	61.	75.8917	{5.50794, 180.553}	92.7538	0.487984	newly	26.	49.62	{5., 116.}	137.769	0.463627	Reff	1.67178	1.57177	{0.995757, 2.21495}	25.1614	ar	754.	670.15	{179., 914.}	28.4231
